# Conducting research in individual patients: lessons learnt from two series of N-of-1 trials

**DOI:** 10.1186/1471-2296-7-54

**Published:** 2006-09-19

**Authors:** Anke CM Wegman, Daniëlle AWM van der Windt, Wim AB Stalman, Theo PGM de Vries

**Affiliations:** 1 Department of Pharmacology/Pharmacotherapy, VU University medical center, Van der Boechorststraat 7, 1081 BT Amsterdam, The Netherlands; 2 EMGO Institute and Department of General Practice, VU University medical center, Amsterdam, The Netherlands; 3 Primary Care Sciences Research Centre, Keele University, Keele, Staffordshire ST5 5BG, UK; 4 EMGO Institute and Department of General Practice, VU University medical center, Amsterdam, The Netherlands; 5 Department of Pharmacology/Pharmacotherapy, VU University medical center, Amsterdam, The Netherlands

## Abstract

**Background:**

Double-blind randomised N-of-1 trials (N-of-1 trials) may help with decisions concerning treatment when there is doubt regarding the effectiveness and suitability of medication for individual patients. The patient is his or her own control, and receives the experimental and the control treatment during several periods of time in random order. Reports of N-of-1 trials are still relatively scarce, and the research methodology is not as firmly established as that of RCTs. Recently, we have conducted two series of N-of-1 trials in general practice. Before, during, and after data-collection, difficulties regarding outcome assessment, analysis of the results, the withdrawal of patients, and the follow-up had to be dealt with. These difficulties are described and our solutions are discussed.

**Discussion:**

To prevent or anticipate difficulties in N-of-1 trials, we argue that that it is important to individualise the outcome measures, and to carefully consider the objective, type of randomisation and the analysis. It is recommended to use the same dosages and dosage forms that the patient used before the trial, to start the trial with a run-in period, to formulate both general and individualised decision rules regarding the efficacy of treatment, to adjust treatment policies immediately after the trial, and to provide adequate instructions and support if treatment is adjusted.

**Summary:**

Because of the specific characteristics of N-of-1 trials it is difficult to formulate general 'how to do it' guidelines for designing N-of-1 trials. However, when the design of each N-of-1 trial is tailored to the specific characteristics of each individual patient and the underlying medical problem, most difficulties in N-of-1 trials can be prevented or overcome. In this way, N-of-1 trials may be of help when deciding on drug treatment for individual patients.

## Background

In medical care, deciding on the optimal treatment for individual patients is one of the main concerns of the physician. Scientific evidence, recommendations from guidelines, medical expertise, patient preferences, and personal experiences all contribute to this decision process. Randomised controlled trials (RCTs) are generally considered to provide the strongest evidence for the efficacy of treatment. However, RCTs are designed to estimate an average treatment effect in a specific population [1]. In daily practice the physician has to determine the extent to which this average effect will apply to an individual patient. For instance, a patient who consults a physician may not be of a similar age, may have additional co-morbidity and medication, or may be interested in a different outcome compared to the subjects studied in the related RCT.

Usually, when a physician doubts the applicability of treatment recommendations (derived from RCTs) to a specific patient, the trial and error method is used ('trial of treatment') [2]. This means that a particular drug will be prescribed, and, subsequently, continued if considered effective, or changed if considered not beneficial. This decision may be strongly influenced by expectations and preferences of both patient and physician. N-of-1 trials provide more objective evidence of individual benefit or harm, while increasing the patient's involvement in the management of his or her disease [3]. In contrast to RCTs, N-of-1 trials do not assess what is best on average for a whole population, but what is best for an individual patient. The patient is his or her own control, and receives the experimental and the control treatment during several periods of time in random order. If possible, the patient, the physician and the researcher are blinded for the sequence of treatments [4]. Therefore, it is reasonable to argue that N-of-1 trials may help physicians to provide better care. However, it is impossible and undesirable to tackle each treatment problem with an N-of-1 trial. Firstly, there should be considerable doubt about the treatment policy. Secondly, the disease or complaint has to be chronic or recurrent, or drugs need to be prescribed for a long period of time or for frequently repeated periods of time. Finally, treatment effects should have a rapid onset and stop acting soon after discontinuation [4].

Reports of N-of-1 trials in general practice are still relatively scarce, and the research methodology is not as firmly established as that of RCTs. Recently, we have conducted two series of N-of-1 trials (Table 1), one in patients with osteoarthritis of the hip or knee (series A), and one in long-term users of temazepam (series B) [5,6]. Before, during, and after data-collection, a number of difficulties had to be dealt with. The aim of this paper is to describe the difficulties we encountered during these series and to discuss our solutions. Successively, difficulties with regard to the outcome assessment, the analysis of the results, the withdrawal of patients, and follow-up will be discussed. We used our own experiences, and searched the literature (Medline 1966 until now, using the search term "N-of-1 trial") to incorporate suggestions made by other researchers regarding the methodology of N-of-1 trials.

**Table 1:** Details of two series of N-of-1 trials conducted by our research group

	Series A [3]	Series B [4]
Setting	General practice	General practice
Subjects	Patients who had been taking NSAIDs regularly in the treatment of pain and disability related to osteoarthritis of the hip or knee.	Long-term users of temazepam (10 or 20 mg) for sleep disturbances.
Objective	1) Is paracetamol as effective as NSAIDs? 2) Is medication-use influenced by presenting the personal results to each individual patient?	1) Is placebo as effective as temazepam (10 or 20 mg), or, in some patients, is 10 mg temazepam as effective as 20 mg temazepam? 2) Is medication-use influenced by presenting the personal results to each individual patient?
Primary outcome measures	1) individual main complaints 2) intensity of pain	1) individual main complaint 2) time to fall asleep
Treatment pairs	2 weeks of paracetamol and 2 weeks of NSAIDs	1 week of placebo and 1 week of 10 mg temazepam, or 1 week of placebo and 1 week of 20 mg temazepam, or 1 week of 10 mg temazepam and 1 week of 20 mg temazapam
Total trial period	20 weeks (5 pairs of treatment periods)	10 weeks (5 pairs of treatment periods)
Sequence of treatments	Randomisation within each treatment pair	Randomisation within each treatment pair
Blinding	The patient, the investigator and the GP were blinded for the sequence of treatments.	The patient, the investigator and the GP were blinded for the sequence of treatments.

## Discussion

### Outcome assessment

#### Problem: which outcome measures should be used?

In a series of N-of-1 trials it seems logical to use the same outcome measures for all patients within one series (as in an RCT). However, since every patient is analysed separately in N-of-1 trials, the outcome measures do not have to be the same for all patients in a series. It is more important that the outcome measures are relevant for each individual patient [7]. On the other hand, in a series of N-of-1 trials, it is of interest to demonstrate variation (heterogeneity) in the results across patients. Therefore, the outcome measures for the different patients should be comparable.

#### Measures taken: outcome measures were individualised

Using the methods proposed by Guyatt et al. [4,8] and Beurskens et al. [9], all patients from series A were asked before the start of their trial to identify their most important problems regarding osteoarthritis. Every day each patient scored the severity of these problems (four to eight items) on a 7-point ordinal scale ranging between 0 = no problem at all and 6 = very severe problem. Examples of such problems were: pain in the knee when going up and down stairs, pain in the knee when lifting shopping bags, stiffness of the knee when getting out of bed. As all items were scored on a similar scale (7-point ordinal scale), differences in the effect of the treatment on the severity of the individual complaints could be compared across patients. Outcome measures were also individualised for series B, in which the patients could select their main problem among a set of questions regarding quantity or quality of sleep.

#### Results of measures

Because we used individualised outcome measures, we were able to formulate treatment recommendations based on the results of patient-specific outcomes. In addition, the patients found it easy to answer questions reflecting their main problems, and completion rates were high. For all patients who finished their trial the completion rate for the main problems was at least 99% in series A, and 82% to 100% in series B. Furthermore, using the same 7-point scale for each problem enabled us to study heterogeneity among patient outcomes. The results ranged from no difference to large differences in favour of non-steroidal anti-inflammatory drugs (NSAIDs) compared to paracetamol in series A, and similarly in series B from no difference to large differences in favour of temazepam compared to placebo or a lower dosage of temazepam.

#### Recommendations

Identification of the main complaint in each patient, yet using the same scale to assess the outcomes for all patients in a series, enables the investigator to present each patient with individualised results and treatment recommendations and, simultaneously, to make comparisons of the results across patients. This approach has been used sucessfully in other N-of-1 trials, investigating the effectiveness of theophylline for irreversible chronic airflow limitation [10] or the efficacy of quinine in leg cramps [11]. Alternatively, patients participating in N-of-1 trials have been asked to give a global assessment of the effectiveness of the medication, and indicate a preference after each treatment pair [12]. Such an assessment is patient centred, and can also take into consideration both beneficial and adverse effects of treatment.

### Analysis of results

#### Problem: how should the outcomes of the N-of-1 trials be analysed?

**Figure 1:**
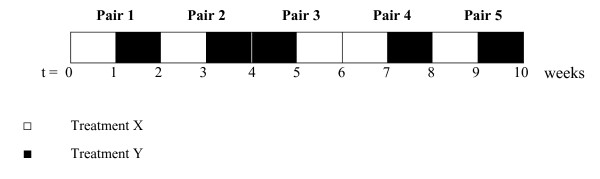
Example of randomisation schedule for one patient receiving 5 pairs of treatment, each consisting of one week of treatment X and one week of treatment Y.

In both our series, the N-of-1 trials consisted of five pairs of treatment periods. Each patient received during one period of each pair the active treatment (treatment X), and during the other period the control treatment or placebo (treatment Y). For each patient, the sequence of treatments (XY or YX) was randomised within each pair of treatment periods (Figure 1).

Since N-of-1 trials are meant to evaluate individual treatment effects (and not to estimate the average effect in a larger population), results are analysed for each patient separately. The possibilities of statistical testing for significance, however, are limited, but in some studies the paired t-test has been used [8,13,14]. The disadvantage of this test is that it assumes a normal distribution of the data, and independence of data from treatment periods. The problem of autocorrelation (i.e. the data are not independent) can be solved by using the average of the data in each treatment period instead of all individual data [4], while non-parametric tests can be applied to tackle violations of the normality assumption. Furthermore, time-series analysis can sometimes be used. In time-series analysis, data over time are compared for separate treatment periods. Time-series analysis is appropriate when there is serial dependency in the data (i.e. successively measured data are significantly correlated). One limitation is that time-series analysis requires a large number of observations in each treatment period. As N-of-1 trials are often designed with relatively short treatment periods, time-series analysis is not really appropriate [15].

Also, non-parametric tests, such as the sign test or the randomisation test can be used. These tests do not require a normal distribution of the data. The disadvantage of the sign test is that it lacks power. At least five pairs of treatment periods are necessary before the significance level of 0.05 can be reached (1/2^5^) [4], irrespective of the magnitude of difference between the treatments. This is the reason why we used five pairs of treatment periods in both our series. With the randomisation test, the power can be increased by using less restricted randomisation schedules [16,17]. For example, if 5 of the 10 periods in our N-of-1 series had been randomly assigned (without restrictions) to treatment X and the other five to treatment Y, the smallest possible p-value would have been 1/252 = 0.004 (=1/number of randomisation possibilities, in this case 1/[10!/5!5!] = 1/252). In case of four periods of treatment X and four periods of treatment Y, this would still be 0.014 (1/[8!/4!4!]). However, restrictions are often necessary to ensure, for example, that the active treatment periods are not all concentrated at the end of the trial, while it could be possible that the patient improves spontaneously [17].

In our series, however, we did not investigate whether one treatment was better than the other, but whether one treatment was equally effective as the other. In other words, the trials we conducted were equivalence trials, rather than superiority trials, which meant that conventional significance testing could not be used for analysis [18-20]. Since 'no difference' between two different treatments cannot be proven, an equivalence range is defined before the start of the trial, i.e. a range of differences between treatments that are considered to be of no clinical importance. If the confidence interval of the difference between treatments lies entirely within this equivalence range, equivalence may be concluded with only a small probability of error [18]. To test equivalence a large number of data are required: the smaller the equivalence range, the more data are needed [18,21,22]. As stated above, the number of observations are often very small in N-of-1 trials, and equivalence testing may not be feasible.

#### Measures taken: computation of median differences in outcomes, and definition of cut-off points

For both series, we decided to define (a priori) cut-off points for a minimal important difference on each primary outcome. In this way we could distinguish between equivalence of effects, small, or large differences in the effectiveness of medication. Given the non-normal distribution of our data, we calculated differences in median scores between the two treatments for each outcome measure. For example, for series B, a median difference in the time to fall asleep of at least 30 minutes in favour of temazepam was considered to be a large effect, 5 to 30 minutes a small effect, and <5 minutes no effect of temazepam.

#### Recommendations

When setting up an N-of-1 trial the investigator should carefully consider the objective of the trial. If the aim is to evaluate whether another type of medication is more effective than current treatment, the trial can be designed as a superiority trial, and conventional tests of significance may be applied during the analysis. If, however, the aim is to confirm the equivalence of two (or more) treatments (which is the case in all efforts to reduce or stop medication), the N-of-1 trial is an equivalence trial, and requires a different method of analysis, with a strong emphasis on the definition of a minimal important difference, and sufficient observations to enable equivalence testing.

Several other researchers have formulated cut-off points or defined responders and non-responders according to the number of pairs in which a clear difference between treatments was seen [23-26]. Patient preferences can be used to determine the outcome of an N-of-trial, with superiority of a drug established if – in a pre-determined proportion of treatment pairs – the patients favours one treatment over the other. In some studies researchers have used this method to allow for a variable number of treatment pairs among patients, with a trial being stopped early in case of strong preferences by either physican or patient [10].

More recently, hierarchical Bayesian methods have been proposed for the statistical analysis of series of N-of-1 trials [27-29]. The objective of this approach is to use individual patient assessments to obtain an overall population estimate of treatment effectiveness that takes into account both the magnitude of the effect, as well as the heterogeneity in individual treatment responses [28]. Bayesian methods have the advantage of allowing for the introduction of co-variates in the model, and for embodying prior information [27]. Therefore, information acquired from previous patients will aid the interpretation of subsequent N-of-1 trial results [27,28]. As the understanding of treatment responses and precision of estimates improve over time, N-of-1 trials may need less cross-overs (treatment pairs) to obtain the same amount of information, or even become unnecessary [28].

### Withdrawal

#### Problem: withdrawal from the trials

Six of the 13 patients (46%) in series A, and three of the 15 patients (20%) in series B, did not complete their trial period. The reasons for withdrawal (as given by the patients) are summarised in Table 2.

**Table 2:** Reasons for withdrawal from the trials

Reasons for withdrawal	No. of patients in series A	No. of patients in series B
Perceived lack of efficacy	4	2
Perceived side effects	1	1
Duration of trial period too long	1	-

Series A: NSAIDs or paracetamol for osteoarthritis

Series B: reduction of temazepam for sleep disturbances

##### Perceived lack of efficacy

Four patients in series A did not finish their trial because of perceived lack of efficacy. Three of these four patients received dosages of NSAIDs during their trial that were lower than they had taken before the trial. The other patient had taken additional paracetamol/codeine before the start of her trial, but was asked not do so during the trial. These findings suggest that the withdrawal of these patients was due to subtherapeutic dosages of medication during their trials. In series B, two patients did not complete their trial because of perceived lack of efficacy. The participants were aware that they would receive placebo or a lower dosage of temazepam half of the time, and may have expected poor results. These expectations may have contributed to a perceived lack of efficacy.

##### Perceived side effects

In both series one patient withdrew because of perceived side effects. Before the start of the trial the patient who withdrew from series A was used to taking diclofenac tablets. To ensure blinding during the trial she received identical capsules containing either diclofenac and placebo, or paracetamol. As the participant reported abdominal complaints during both periods it is possible that these symptoms were caused by the gelatine capsules. Similarly, a patient in series B received medication in a different dosage form (tablets instead of temazepam capsules). This patient reported nausea, and withdrew after a few days. The reports of nausea and abdominal complaints may also have been the result of pure coincidence, or perhaps, be related to concerns regarding the effects of taking medication in another form.

##### Duration of the trial

In series A the duration of the trial was 20 weeks, and in series B it was 10 weeks, which made it easier for the patients in series B to complete their trial. In series A, one patient withdrew because the study took too long and because of coexisting symptoms (back pain). None of the patients in series B withdrew because the trial took too long.

#### Recommendations

A number of measures can be taken to prevent withdrawal due to subtherapeutic dosages or (perceived) side effects. Firstly, the drug can be prescribed in the same dosage and form as the patient is used to. Secondly, the trial can be started with a run-in period, during which the patient receives the drug in the dosage and form in which it will be prescribed during the trial. In this way, the patient can withdraw from the trial at an early stage, or the medication can be adjusted before the data-collection actually starts. Finally, if blinding is difficult because of insurmountable differences in the form, size, colour, smell or taste of the medication, the double-dummy design can be used. In a double-dummy design the patient receives active treatment X and placebo treatment Y during one period, and placebo treatment X and active treatment Y during the other. However, this will increase the number of tablets that need to be taken, possibly leading to non-compliance or withdrawal [23].

An alternative method to prevent withdrawal due to expectations of poor results is to keep the patients completely unaware of the dosages. However, this may not receive ethical approval. Furthermore, to prevent withdrawal due to the length of the trial, treatment periods can be kept as short as possible. Such a decision will affect the power of the trial. In a commentary on an N-of-1 trial in a pregnant woman with nausea and morning sickness [30-32] Campbell discussed the length of treatment periods [33]. While the total trial period should not be too long [34] the separate treatment periods should be long enough to achieve and detect a clinically important treatment effect [31].

Withdrawal rate in series of N-of-1 trials conducted in primary care range between 12 and 40%, with similar reasons for discontinuing participation [2,11-13,24-26,29]. Nikles et al. proposed that withdrawal rates can be thought of as a general measure of compliance (or adherence), which is often not as good as clinicians believe. Non-adherence rates of around 50% have been reported for several chronic conditions [2].

### Follow-up

#### Problem: reverting to the same medication as before the N-of-1 trial

After the completion of a trial we visited participants at home to discuss their results. Subsequently, patients were asked about their intentions regarding future medication intake for osteoarthritis (series A) or sleep disturbances (series B), and general practitioners (GPs) were informed about these intentions. However, three months after the completion of series A, four of the six patients who intended to switch to paracetamol were taking NSAIDs for osteoarthritis, and one patient was taking both paracetamol and NSAIDs. In series B, two of the nine patients who intended to reduce or stop their temazepam intake had reverted to their previous dosage. The reasons for reverting to the same medication are summarised in Table 3.

**Table 3:** Follow-up: Reasons for reverting to the same medication as before the N-of-1 trial

Reasons for reverting in series A
• Perceived lack of efficacy of paracetamol
• Deterioration of osteoarthritis
• Misunderstanding regarding paracetamol dosage
• Preference of small tablets (diclofenac) over larger ones (paracetamol)
• Patient found it a waste to throw away the NSAIDs she still had in her possession
Reasons for reverting in series B
• Dosage of temazepam had not been adjusted after the N-of-1 trials
• Patient sees no disadvantages in taking temazepam

Series A: NSAIDs or paracetamol for osteoarthritis

Series B: reduction of temazepam for sleep disturbances

##### Patients were not sufficiently instructed, coached and/or monitored

One patient from series A indicated that she had reverted to taking NSAIDs because the paracetamol was not sufficiently effective. However, instead of taking 1000 mg paracetamol three times a day, the patient had been taking 500 mg twice a day. After solving this misunderstanding, the patient agreed to start taking paracetamol again. Another patient considered it a waste to throw away the NSAIDs and wanted to finish her supply before starting the paracetamol treatment. In one patient from series B the dosage of temazepam had not (yet) been reduced. Since the end of the trial, the patient had not seen the GP and neither the patient nor the GP had taken any action to adjust the treatment policy. Obviously, these three patients were not sufficiently instructed by the researcher or sufficiently coached and monitored by the GP.

##### Motivation to adjust treatment

In one patient from series B temazepam had little effect on the quality or quantity of sleep, but the patient saw no disadvantage in taking temazepam and decided to take it just as frequently as before the trial.

#### Recommendations

Only few reports on N-of-1 trials have discussed the long-term effects of participating in an N-of-1 trial [10-12]. Both Woodfied et al. [11] and Mahon et al. [10] commented on the fact that a considerable number of participants decide to resume or continue the use of active medication, despite results of the trial indicating a lack of effectiveness. Some of these decisions were explained by the fact that participants returned to their own non-study physician after completion of the trial. To prevent patients from reverting to previous treatment, a number of measures can be taken: (1) before the start of the trial the patients should be well informed about the objectives and, especially, the possible consequences of the trial, (2) the GP (together with the researcher) should discuss the results with the patient and adjust (if appropriate) the treatment policy immediately, (3) the patients should be well instructed and encouraged to follow treatment recommendations, and (4) monitoring may be needed for some time. Furthermore, before the start of a trial, the patient's reasons for participation should be investigated. Patients should be willing to question their current treatment, and adjust their treatment, if necessary [35]. We suggest to define a minimal important difference for each patient before the start of the trial. This will establish individually relevant decision rules with clear agreements regarding the interpretation of results and decisions regarding the future treatment policy.

## Summary

Given the importance of adjusting the design of an N-of-1 trial to the specific characteristics of each individual patient, it is difficult to formulate general 'how to do it' guidelines for N-of-1 trials. However, when the design of each N-of-1 trial is tailored to the specific characteristics of each individual patient and the underlying medical problem, most difficulties in N-of-1 trials may be prevented or overcome. In our opinion, it is important to carefully consider the objective of the trial, and, with that, the randomisation schedule and the type of analysis. Furthermore, it is important to individualise outcome measures, so that they are relevant for each patient. We recommend that dosage and form of medication is the same as before the trial, and that the trial starts with a run-in period. General decision rules regarding the efficacy of treatment may help to demonstrate variation across patients, but we also recommend to formulate individualised decision rules for the future use of medication before the start of a trial. Finally, patients need to be provided with adequate instructions and support after adjustments of treatment.

### Usefulness and implementation of N-of-1 trials

The value of N-of-1 trials for making decisions in individual patients, through bridging the gap between research and practice, allowing for an individual approach, and incorporating patient values, has been well-accepted [36]. Results of randomised N-of-1 trials have even been placed in the top of the hierarchy of evidence [37]. However, few studies have evaluated their usefulness in clinical practice, or assessed potential barriers to more widespread implementation of this approach. Wide experience has been gained in Australia, where a national N-of-1 service has been implemented. The service has been promoted through clinical meetings, newsletters, websites, and the media [26]. Several large series of N-of-1 trials have been undertaken [26,29,38]. Qualitative research showed that patients were generally very satisfied with the N-of-1 trial process. Their participation led to increased awareness and understanding of their condition, and its management, resulting in a sense of empowerment and control [2]. The effort of setting up an N-of-1 trial in primary care, however, is substantial [3], especially outside the setting of an N-of-1 trial service. It includes the costs of paperwork and consent forms, arranging identical placebo's from a pharmacy, designing and printing diaries, statistical analysis, and time spent informing and instructing patients.

The ultimate test of the usefulness of N-of-1 trials in clinical practice is to compare the outcome of N-of-1 trials in terms of costs and patient benefits to usual care in a randomised design. Two such randomised trials have been undertaken. Mahon et al. [10] evaluated whether patients with irreversible chronic airflow limitation who were prescribed theophylline guided by N-of-1 trials had better outcomes than patients treated according to standard practice. The use of N-of-1 trials did not yield important advantages over standard practice. Pope et al. compared the efficacy and cost-effectiveness of placebo-controlled N-of-1 trials with diclofenac/misoprostol to standard care among patients with osteoarthritis who were uncertain that NSAIDs would be helpful [12]. The N-of-1 trials yielded slightly better improvements than standard care, but the difference was not statistically significant. Both trials showed that the costs of N-of-1 trials were considerably higher than those of standard care, due to the costs associated with the production of placebo's, and time spent by nurses, physicians, and patients. These were the first trials that formally evaluated the cost-effectiveness of implementing N-of-1 trials in clinical practice. The number of patients involved was rather small, and follow-up was limited to six or 12 months. Additional research among patients with different types of medical problems is needed to firmly establish the value and need of the N-of-1 approach in primary care.

## Competing interests

The author(s) declare that they have no competing interests.

## Authors' contributions

All four authors discussed with eachother the themes of this manuscript and made substantial contibutions to the design of the manuscript. AW and DvdW drafted the manuscript and the other authors revised it critically. All four authors have read and approved the final version of the paper.

## Pre-publication history

The pre-publication history for this paper can be accessed here:


